# Magnetic-Field-Induced Suppression of Jahn-Teller Phonon Bands in (La_0.6_Pr_0.4_)_0.7_Ca_0.3_MnO_3_: the Mechanism of Colossal Magnetoresistance shown by Raman Spectroscopy

**DOI:** 10.1038/s41598-019-39597-1

**Published:** 2019-02-20

**Authors:** S. Merten, O. Shapoval, B. Damaschke, K. Samwer, V. Moshnyaga

**Affiliations:** 1 I. Physikalisches Institut, Georg-August-Universität Göttingen, Friedrich-Hund-Platz 1, D-37077 Göttingen, Germany; 2 IIEN, Academy of Sciences of Republic Moldova, Strada Academiei 3/3, MD-2028 Chisinau, Republic of Moldova

## Abstract

A long-standing issue in the physics of the colossal magnetoresistance is the role of electron-phonon coupling, which manifests itself as Jahn-Teller polarons. The origin and architecture of polarons makes it possible to study their behavior by Raman spectroscopy, which allows to analyze the polaronic behavior in an applied magnetic field. We performed magnetic-field-dependent Raman spectroscopy on thin films of (La_0.6_Pr_0.4_)_0.7_Ca_0.3_MnO_3_ in a range of *H* = 0–50 kOe and compared the obtained Raman spectra with the magnetic field behavior of the electrical resistivity. In the vicinity of the Curie temperature, *T*_C_ = 197 K, the intensity of the Jahn-Teller stretching mode at 614 cm^−1^ and of the bending mode at 443 cm^−1^ was found to be suppressed and enhanced, respectively. This observed behavior has a remarkable similarity with the field and temperature dependence of the colossal magnetoresistance in (La_0.6_Pr_0.4_)_0.7_Ca_0.3_MnO_3_. Our work provides direct evidence that the reduction of the amount of Jahn-Teller polarons at the phase transition is the main mechanism underlying the colossal magnetoresistance.

## Introduction

The rediscovering of the colossal magnetoresistance (CMR), i.e. a drastic decrease of electrical resistance in an applied magnetic field, in thin mixed-valence manganite films^1,2^ initiated an enormous boom of fundamental and applied research. For over twenty years, the long-standing important issue in CMR physics is the role of electron-phonon coupling and the mechanisms of electron-lattice correlations, controlling the coupled magnetic and metal-insulator transitions. In systems with strong electron-phonon coupling^3^, like (La_1−y_Pr_y_)_0.7_Ca_0.3_MnO_3_, a pronounced nm-scale coexistence with ferromagnetic (FM) metallic and charge-ordered insulating (COI) phases^4^ and extremely large CMR (CMR ≈ 10^5^–10^7^%) were observed. In the parent compound LaMnO_3_ (LMO), the Jahn-Teller (JT) effect solely determines the structure and magnetism by means of a cooperative ordering of Jahn-Teller distorted MnO_6_ octahedra. In doped manganites, like (La_0.6_Pr_0.4_)_0.7_Ca_0.3_MnO_3_ (LPCMO), only short-range static JT distortions, dubbed as correlated polarons^5^ (CPs), exist and contribute to the phase separation. CPs are viewed as charge/orbital ordered (COO) lattice superstructures of the CE type and with antiferromagnetic (AFM) correlations at a length scale, *d*_CP_ ≈ 1–2 nm, revealed by neutron and x-ray scattering^6,7^. Recently, we have shown that even a tiny amount of CPs plays a key role in CMR as they are able to mediate an intrinsic AFM coupling between the nm-size FM domains, yielding an increase of the resistivity at the metal-insulator transition for antiparallel orientations of FM nano-domains at the coercive field *H*_c_^8^. Furthermore, *Michelmann et al*. observed a softening of the bulk modulus near *T*_C_, which can be attributed to the strong electron-phonon coupling in the (La_0.6_Pr_0.4_)_0.7_Ca_0.3_MnO_3_ (LPCMO) system and thus, the presence of correlated polarons^9^.

The origin and architecture of correlated polarons as short-range JT distortions makes it possible to probe them by Raman spectroscopy and to monitor their behavior across the phase transition. For the orthorhombic structure, e.g. LMO, the two high frequency modes at 490 cm^−1^ and 611 cm^−1^ are of particular interest, since they are directly related to the coherent JT distortion in the system^10^. These Raman features are forbidden in rhombohedral manganites, possessing no static JT distortions. Upon doping with divalent alkaline earth elements, like Ca^2+^, an oxygen disorder is introduced into the MnO_6_ octahedral network of LMO and thus, instead of a coherent, the incoherent JT distortions are formed. This leads to a broadening of the JT modes due to a disorder-induced phonon scattering and can be qualitatively described by means of the phonon density of states (PDOS)^11^. Temperature-dependent Raman spectra of La_0.7_Ca_0.3_MnO_3_^11,12^ have shown a remarkable interplay between the broad JT stretching mode around 600 cm^−1^ and a sharp bending mode around 438 cm^−1^: the intensity of the former is suppressed and of the latter is increased below *T*_C_. According to the disorder-order scenario of the phase transition, proposed by *Iliev et al*.^11^, the average lifetime, *τ*_h_, of a Mn^3+^ state in the paramagnetic state (*T* > *T*_C_), i.e. the time between two consecutive Mn^3+^–>Mn^4+^ hopping events, is larger than the average lifetime of the JT distortion, *τ*_JT_. Hence, a quasi-static JT distortion develops yielding the broadening of the Raman mode. In the FM metallic state (*T* < *T*_*C*_), in turn, *τ*_h_ ≪ *τ*_JT_, resulting in a suppression of the JT mode, since no more Mn^3+^ ions can exist. The arising sharp bending mode corresponds to a Γ-point phonon of a more ordered structure so that, the insulator-metal and paramagnetic-to-ferromagnetic transition is accompanied by a disorder-order transition in the MnO_6_ octahedral network. To the best of our knowledge, no magnetic-field-dependent Raman studies of the optimally doped CMR materials, e.g. LPCMO, were reported up to now. One can expect that in such a material a similar effect will occur in an applied magnetic field at *T* ≈ *T*_C_. A previous magnetic-field-dependent Raman study was focused on the melting of the long-range-ordered COO insulating phase in La_x_Pr_y_Ca_1−y−x_MnO_3_ (y = 0.6, x = 0.375)^13^. However, in contrast to LPCMO, the magnetic-field-induced melting of the COO phase is accompanied by a structural phase transition from a monoclinic (*P2*_1_*/m*) to the orthorhombic (*Pnma*) structure.

Here, we report a magnetic-field-dependent Raman study in thin films of a classic CMR material (La_0.6_Pr_0.4_)_0.7_Ca_0.3_MnO_3_ and discuss it in terms of the disorder-order phase transition driven by magnetic field. The magnetic-field-induced suppression of the JT mode and an intensity gain of the bending mode was observed in the vicinity of *T*_C_, demonstrating the key role of the correlated JT polarons in the CMR effect.

## Results and Discussion

**Figure 1:**
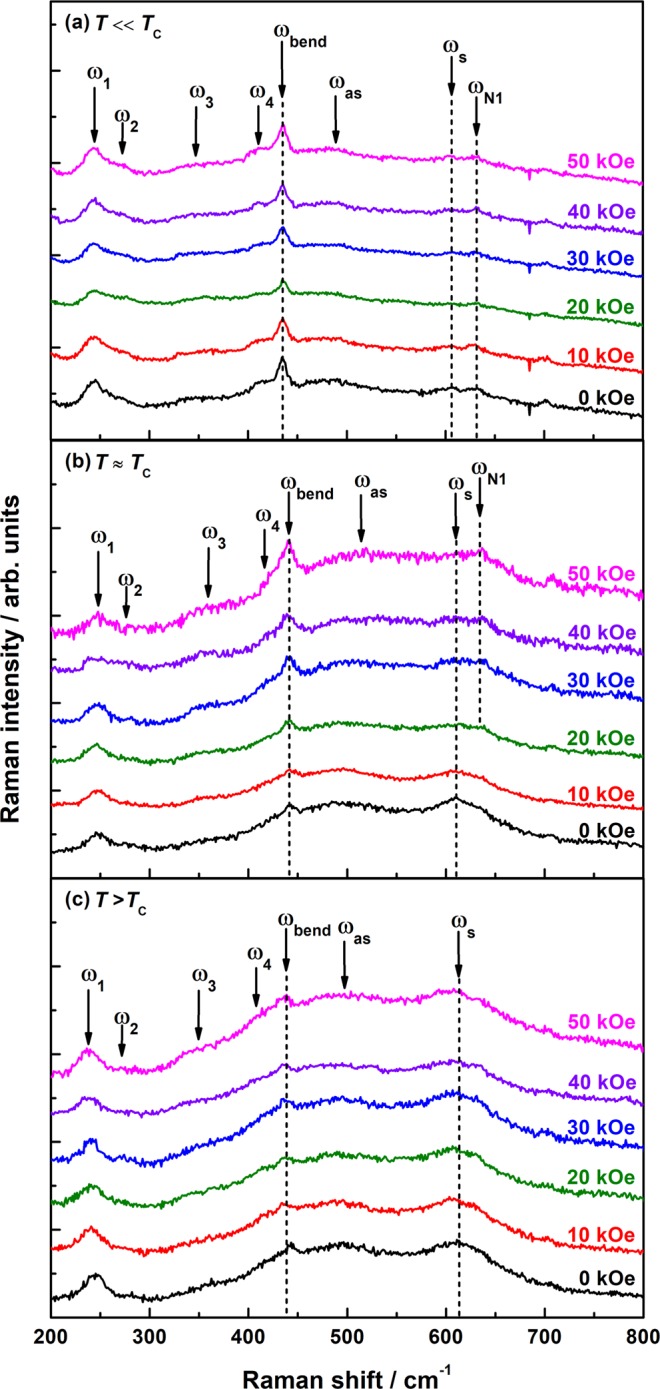
Magnetic-field-dependent Raman spectra of LPCMO measured at different temperatures: (**a**) *T* = 90 K ≪ *T*_C_ (**b**) *T* = *T*_C_ = 195 K (**c**) *T* = 245 K > *T*_C_. The dashed lines indicate the Raman modes of interest, the bending mode *ω*_bend_ and the JT stretching mode *ω*_s_ as well as the extrinsic JT mode *ω*_N1_

**Figure 2:**
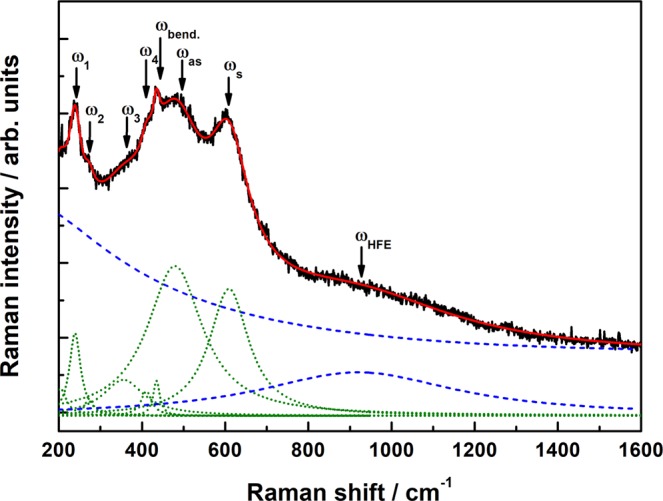
The Raman spectrum at 245 K of LPCMO and the best fit curve (red) are shown. The phonon contribution (dotted green) and the electronic contributions (dashed blue) are shown separately.

Structural, electrical and magnetic characterization of the LPCMO film (see Supplementary Information S1) has revealed a coupled metal-insulator and ferromagnetic-paramagnetic phase transition at *T*_C_ = 197 K. In Fig. 1, the unpolarized Raman spectra, measured in applied magnetic fields, *H* = 0–50 kOe, are shown for (a) *T* ≪ *T*_C_ (b) *T* = *T*_C_ and (c) *T* > *T*_C_. The electronic background continuum was modeled by a collision-limited model^14^ and the phonon peaks were fitted by multiple Lorentzian line shapes. As an example the best fit curve and the different components are shown in Fig. 2 (see also the Supplemental Information S2). The agreement between the experimental Raman spectra and the fitting curves were quite good at all analyzed temperatures and magnetic fields (R^2^ ≈ 0.99). Furthermore, no signs of a Fano line shape were observed at any temperature or applied magnetic field, indicating no interaction between the electronic background continuum and the phonons^15^. The Raman spectra of LPCMO consist of seven phonon modes characteristic for manganites with orthorhombic structure e.g. La_0.7_Ca_0.3_MnO_3_^11^ or undoped orthorhombic LaMnO_3_:^10,16^ rotational modes at 245 cm^−1^ (*A*_g_, *ω*_1_) and 280 cm^−1^ (*A*_g_, *ω*_2_), a displacement of the oxygen ion at 360 cm^−1^ (*B*_2g_, *ω*_3_), bending modes at 424 cm^−1^ (*A*_1g_, *ω*_4_) and 443 cm^−1^ (*B*_2g_, *ω*_bend_) as well as the JT stretching modes at 492 cm^−1^ (*A*_g_, *ω*_as_) and at 614 cm^−1^ (*B*_2g_, *ω*_s_), respectively. The high frequency electronic (HFE) contribution at ≈ 900 cm^−1^ was assigned to the photoionization of small polarons^14^. For *T* > *T*_C_ the stretching mode (*ω*_s_) shows a small shoulder and splits visibly into two modes at 614 cm^−1^ (intrinsic) and 630 cm^−1^ (extrinsic) for temperatures *T* < *T*_C_ and magnetic fields, *H* > 20 kOe, respectively. This additional mode at ≈ 630 cm^−1^ (*ω*_N1_) was also observed in a thickness-dependent Raman study on LaMnO_3_ and is assigned to the film/substrate interface^17^, pointing out its extrinsic origin.

In the following, we will focus on the magnetic field behavior of the stretching mode *ω*_s_ as well as of the bending mode *ω*_bend_, because of their interplay at the temperature-driven phase transition^8,11^. First, we will look at the Raman spectra measured far away from the transition temperature *T*_C_, shown in Fig. 1a,c. In the insulating paramagnetic state, *T* > *T*_C_, an applied magnetic field has no influence on the phonon modes. A similar behavior is observable for temperatures *T* ≪ *T*_C_, i.e. deep in the FM metallic state. Therefore, we conclude that deep in the insulating and metallic state, the phonon and the electron^8^ system of LPCMO is stable against an external magnetic field. In contrast, in the vicinity of *T*_C_ (Fig. 1b), one can see a strong suppression of the JT modes and a less pronounced intensity increase of the bending mode *ω*_bend_ is observable. Note that even at high magnetic fields, i.e. *H* = 50 kOe, remnants of the JT modes are still visible supporting the model of electronic phase separation^4,8,11^. In addition, the above mentioned splitting of the *ω*_s_ mode can also be seen at *T* = *T*_C_ and magnetic fields *H* > 20 kOe. It becomes even clearer due to the progressive suppression of the intrinsic JT mode and the less sensitive field behavior of the extrinsic one.

**Figure 3:**
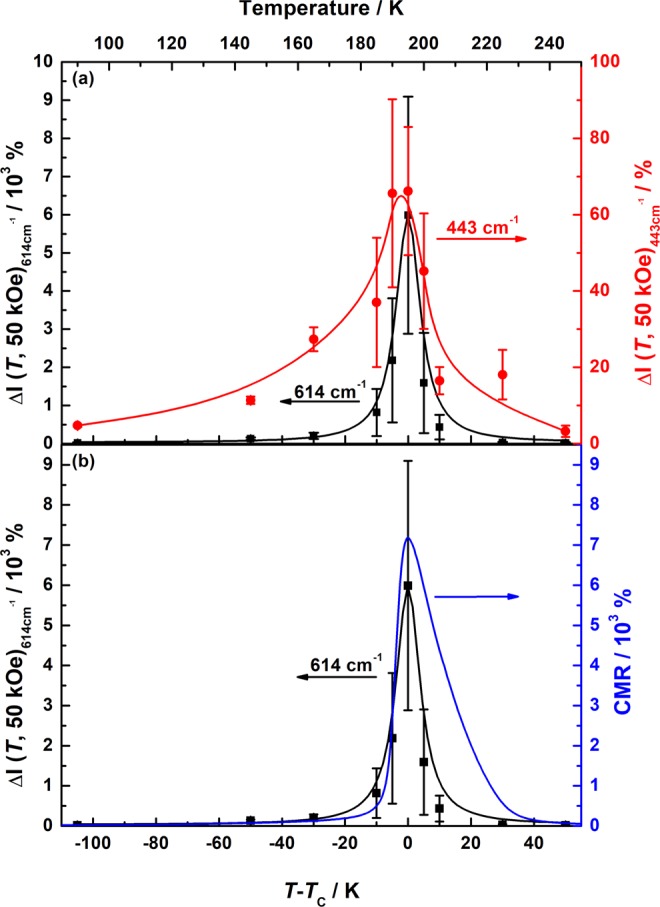
(**a**) The Δ*I*_j_(*T*, 50 kOe) ratios of the JT mode (*ω*_s_, left scale) and the bending mode (*ω*_bend_, right scale) differing about two orders of magnitude (Δ*I*_s_(*T*, 50 kOe) ≈ 6000%, Δ*I*_bend._(*T*, 50 kOe) ≈ 65%). For both modes, Δ*I* develops below *T* = 225 K coinciding with the metamagnetic transition temperature. (**b**) A comparison between the Δ*I*_s_ (left scale) and the CMR ratio (right scale) illustrates their common polaronic nature.

To illustrate the close relation of the JT (*ω*_s_) and bending mode (*ω*_bend_) to the phase transition, one can summarize their field behavior as the relative change of the intensities of the corresponding Raman modes in a magnetic field as a function of temperature, i.e. in a Δ*I*_j_ (*T*, 50 kOe) = 100%|(*I* (50 kOe) – *I* (0 kOe))/*I* (50 kOe)| vs. Δ*T* = *T* − *T*_C_ plot as shown in Fig. 3a. One can see that the Δ*I*_j_ (*T*, 50 kOe) behavior of these modes show a remarkable qualitative and, in the case of *ω*_s_, even a quantitative similarity to the CMR effect (see Fig. 3b). Indeed, the CMR and Δ*I*_j_ (*T*, 50 kOe) are maximal only in the vicinity of *T*_C._ Note that, the intrinsic JT mode shows much higher field sensitivity than the bending mode. The relative change of ω_s_ is negative, but since the Δ*I*_j_ (*T*, 50 kOe) is defined as the absolute value, the sign is not reflected in the Δ*I* (*T*, 50 kOe) value. Quantitatively, the observed Δ*I*_s_ (*T*, 50 kOe) ≈ 6∙10^3^% of the intrinsic JT mode is of the same order of magnitude as the CMR ≈ 7∙10^3^%, whereas Δ*I*_bend_ (*T*, 50 kOe) ≈ 65% is by about two orders of magnitude smaller (see Fig. 3a).

Moreover, one can also see a difference of the linewidth of the Δ*I*_s_ (*T*, 50 kOe) and the CMR: the former shows an extremely sharp maximum close to *T*_C_ and the latter is significantly broader for *T* > *T*_C_. Maybe, this is related to the difference in the probed scale as the CMR was measured on the macroscopic mm-scale, but Δ*I*_s_ (*T*, 50 kOe) was taken locally on the μm-scale. In addition, the probed systems are different, i.e. electric in case of CMR and phononic in Δ*I*_s_ (*T*, 50 kOe). Nevertheless, the comparable Δ*I*_s_ (*T*, 50 kOe) and CMR ratios demonstrate unambiguously the importance of the correlated JT polarons, giving evidence that their reduction is the main mechanism underlying the CMR effect.

**Figure 4:**
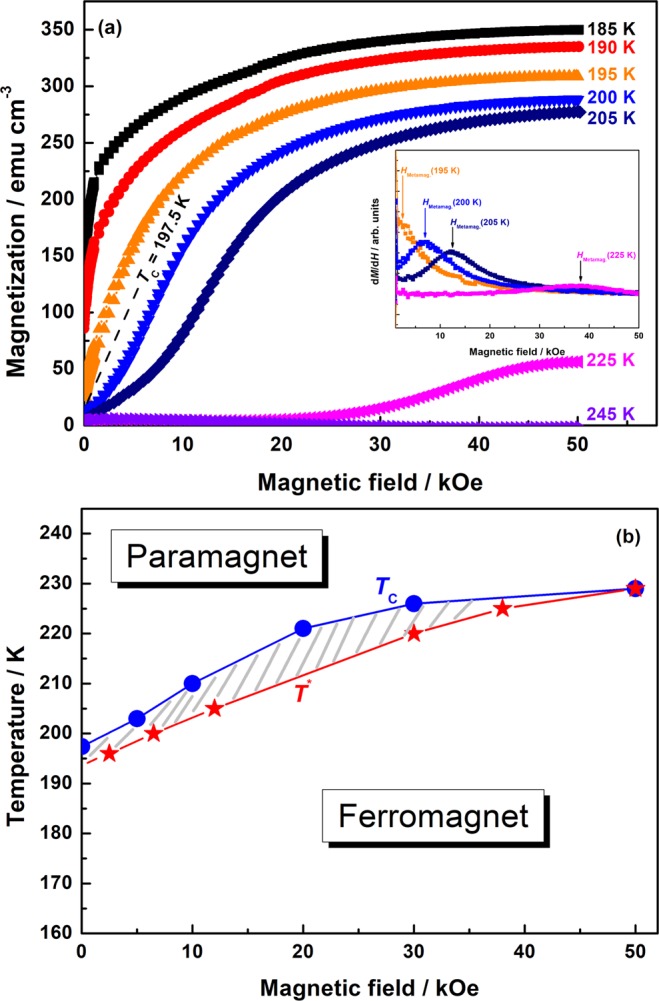
(**a**) Field dependencies of the magnetization measured in the vicinity of *T*_C_, measured for magnetic fields *H* = 0–50 kOe. The inset shows d*M*/d*H* curves for selected temperatures, *T* = 195 K, 200 K, 205 K, 225 K. (**b**) The evaluated *T*-*H* phase diagram with *T*_C_ and T^*^ lines illustrates the existence of an inhomogeneous magnetic state for T^*^ < *T* < *T*_C_, where FM nanodomains are AFM coupled by correlated JT polarons. Their field-induced melting results in a metamagnetic transition, accompanied by Δ*I*_j_(*T*, 50 kOe) and CMR behavior.

The origin of the magnetic-field-dependent Raman behavior and its relation to the magnetic phase transition and the CMR can be illustrated by a *T*-*H* phase diagram, obtained by measuring the magnetization loops *M*(*H*) at different temperatures across the phase transition, shown in Fig. 4a (see also Supplementary Information S3). The obtained *T*-*H* phase diagram, shown in Fig. 4b, reveals two important temperature scales: (1) the *T*_C_(*H*)-line indicating a magnetic field dependence with a saturating value of *T*_C_ (*H* = 50 kOe) = 229 K and (2) the T^*^(*H*)-line separating a mixed state with a metamagnetic transition from a homogeneous FM state, which is developed for *T* < T^*^(*H*). The narrow temperature distribution of the Δ*I*_s_ (*T*, 50 kOe), Δ*T*_ΔI(T, 50 kOe)_ = ±10 K, and the CMR, Δ*T*_CMR_ = ±30 K, around *T*_C_ shows their common origin and location between the T^*^- and *T*_C_ -lines in the *T*-*H* diagram, i.e. within the inhomogeneous magnetic state. Such a complex magnetic behavior close to the 1^st^ order phase transition was introduced in earlier theoretical CMR studies^3,18^ and supported by magnetic^4,8^ and ultrasonic^9^ experiments on LPCMO. An applied magnetic field couples to the short-range-ordered polaronic AFM phase and melts the polaronic phase by aligning the spins parallel to the field, yielding the suppression of the JT modes in the Raman spectra. These changes go along with a drastic increase of the magnetic moment, i.e. a metamagnetic transition, and a reduction of the resistance manifesting as the CMR. Note that, both temperatures, *T*_C_ and T^*^, merge into a common value in the *T*-*H* phase diagram, which is related to the temperature of the charge ordering (CO) transition, *T*_CO_ ≈ 225–230 K, in manganites^4^. Above *T*_CO_, the PM phase remains fully symmetric, since neither CO and nor AFM correlations are energetically more favorable.

As mentioned before, the doping of the parent compound LaMnO_3_ with divalent cations induces an oxygen disorder in the system and thus, in the MnO_6_ network, which results in a broadening of the JT modes^11^. Lowering the temperature below *T*_C_, suppresses the polaronic phase, i.e. the disorder in the system, resulting in an increase of structural order within the MnO_6_ network, reflected by the intensity increase of the bending mode *ω*_bend_. A similar process occurs in an applied magnetic field in the vicinity of *T*_C_. The suppression of the polaronic phase results in a strong suppression of the JT mode (*ω*_s_) and in a more moderate increase of the intensity of *ω*_bend_. This means that the field-induced decrease of disorder close to *T*_C_ does not seem to be instantly converted into the same amount of structural order in the MnO_6_ system. Since the octahedral system remains at a relatively high temperature (*T* = *T*_C_ ≈ 197 K), the disorder-order conversion is relatively ineffective. The reason is, likely, the lifetime *τ*_h_ of a Mn^3+^ state and its strongly non-linear temperature behavior close to *T*_C_^19^. At low temperatures, i.e. *τ*_h_ ≪ *τ*_JT_, the further decrease of *τ*_h_ in an applied magnetic field enables an instantaneous gain in order. Close to the phase transition, *τ*_h_ is comparable to *τ*_JT_ and only a small amount of disorder can be transformed. Since *τ*_h_ < *τ*_JT_, the JT distortion, however, can be significantly reduced resulting in a strong magnetic-field-induced effect for *ω*_s_, but a much smaller for the bending mode *ω*_bend_.

**Figure 5:**
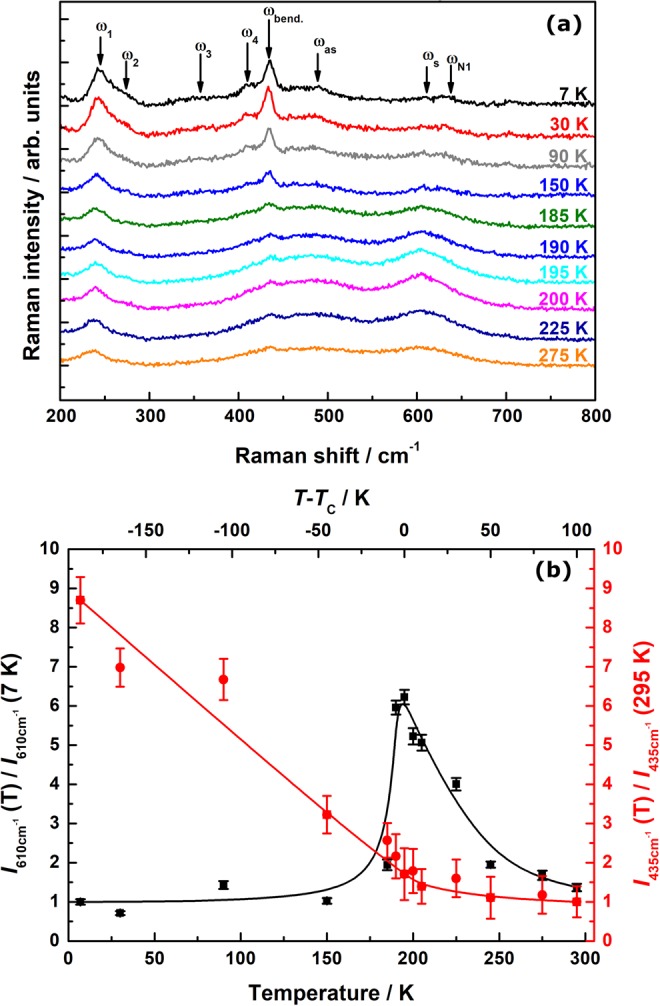
Temperature-dependent Raman spectra of LPCMO (**a**) and the evaluated temperature dependency (**b**) of the intensity of the JT mode *ω*_s_ (610 cm^−1^, black) and of the bending mode *ω*_bend_ (435 cm^−1^, red).

Temperature-dependent measurements at ambient magnetic field, *H* = 0 kOe, show a similar behavior (see Fig. 5). Below *T*_C_, the polaronic phase shrinks rapidly and remains at a constant level, represented by the small intensity of the JT mode *ω*_s_. The bending mode, in turn, gains significant intensity only below *T*_C_, continuously increasing down to 7 K. One could expect a further field-induced increase of the bending mode at *T* = *T*_C_, i.e. higher values of Δ*I*_bend_ (*T*, 50 kOe) for *H* > 50 kOe analogously to its temperature dependence.

In summary, magnetic-field-dependent Raman spectroscopy on a thin LPCMO film in a broad range of temperatures and magnetic fields was carried out. We obtained direct evidence that the reduction of the amount of correlated JT polarons is the main mechanism underlying the CMR effect emphasizing the importance of strong electron-phonon coupling for CMR materials.

## Methods

LPCMO film has been grown by a metalorganic aerosol deposition (MAD) technique^20^ on commercial MgO(100) substrate (*Crystal GmbH*). Acetylacetonates of La, Pr, Ca and Mn were used as precursors. The film was deposited at a substrate temperature *T*_sub_ = 950 °C with a growth rate *v* = 10 nm/min and was cooled down to room temperature in 30 min after deposition. X-ray diffraction in Θ−2Θ Bragg-Brentano geometry with Cu_kα_ radiation and small-angle X-ray reflectivity were performed to characterize the structure and thickness of the film. Magnetic (SQUID MPMS, *Quantum design*) and electrical four-probe characterizations (PPMS, *Quantum design*) were carried out for temperatures *T* = 5–300 K and magnetic fields *H* = 0–50 kOe. Raman measurements were performed in a backscattering-geometry with a continuous-wave Nd:YAG laser (*λ* = 532 nm) by means of a confocal Raman microscope (LabRAM HR Evolution*, Horiba JobinYvon*), equipped with a thermoelectrically cooled charge-coupled device of 1024 × 256 pixels. The laser beam was focused onto the sample surface within a spot size, *d*_spot_ ≈ 1.29 μm. To avoid significant heating of the sample, the laser power was kept at *P* = 2.9 mW (see Supplementary Information S2). The Raman setup was optically coupled to a continuous-flow He cryostat (Microstat MO, *Oxford Instruments*) to measure Raman spectra for temperatures, *T* = 90 K–245 K, and magnetic fields, *H* = 0–50 kOe. A superconducting solenoid produces a magnetic field perpendicular to the sample surface (see Supplementary Information S4). No correction of the spectrometer response was made.

## Supplementary information

Supplementary Information

## Data Availability

The datasets generated during and/or analysed during the current study are available from the corresponding author on reasonable request.
