# Supplementary material for: Magnetic-Field-Induced Suppression of Jahn-Teller Phonon Bands in (La0.6Pr0.4)0.7Ca0.3MnO3: the Mechanism of Colossal Magnetoresistance shown by Raman Spectroscopy
Source: Sci Rep. 2019 Feb 20;9:2387. doi: 10.1038/s41598-019-39597-1 (PMC6382866; doi:10.1038/s41598-019-39597-1)
Supplement: Supplementary file 1 — Supplementary Information [file 41598_2019_39597_MOESM1_ESM.pdf]

Supplementary Information for "Magnetic-Field-Induced Suppression of Jahn-Teller Phonon Bands in $(La_{0.6}Pr_{0.4})_{0.7}Ca_{0.3}MnO_3$: the Mechanism of Colossal Magnetoresistance shown by Raman Spectroscopy"

S. Merten[1] *, O. Shapoval[2], B. Damaschke[1], K. Samwer[1] and V. Moshnyaga[1]

[1] *I. Physikalisches Institut, Georg-August-Universität Göttingen, Friedrich-Hund-Platz 1, D-37077 Göttingen, Germany*

[2] *IIEN, Academy of Sciences of Republic Moldova, Strada Academiei 3/3, MD-2028 Chisinau, Republic of Moldova*

Herewith, we provide all additional information regarding the characterization of the LPCMO film (S1), the evaluation of the Raman spectra (S2), deduction of the *T*-*H* phase diagram (S3) and a sketch of our experimental setup (S4).

S1. Structural, electrical and magnetic characterization

X-ray diffraction (XRD) and X-ray reflectometry (XRR) structural characterization of our LPCMO film demonstrates an out-of-plane epitaxial grown film on the MgO(100) substrate with a pseudo-cubic lattice constant, a = 3.871 Å, and a thickness, d = 66 nm. Electric and magnetic measurements display coupled insulator-metal and paramagnetic-ferromagnetic transitions with a transition temperature $T_{MI} \approx T_C \approx 197$ K (see Fig. S1.1). In Fig. 1.2, we present the $R(T)$ curves for different applied magnetic fields, H = 0 kOe, 10 kOe, 30 kOe and 50 kOe.

Fig. S1.1: (a) XRD and XRR pattern and (b) $R(T)$ and $M(T)$ curves of the thin LPCMO film. The LPCMO film shows out-of-plane epitaxial growth with a pseudo-cubic lattice constant, a = 3.871 Å and a transition temperature $T_{MI} \approx T_C \approx 197$ K.

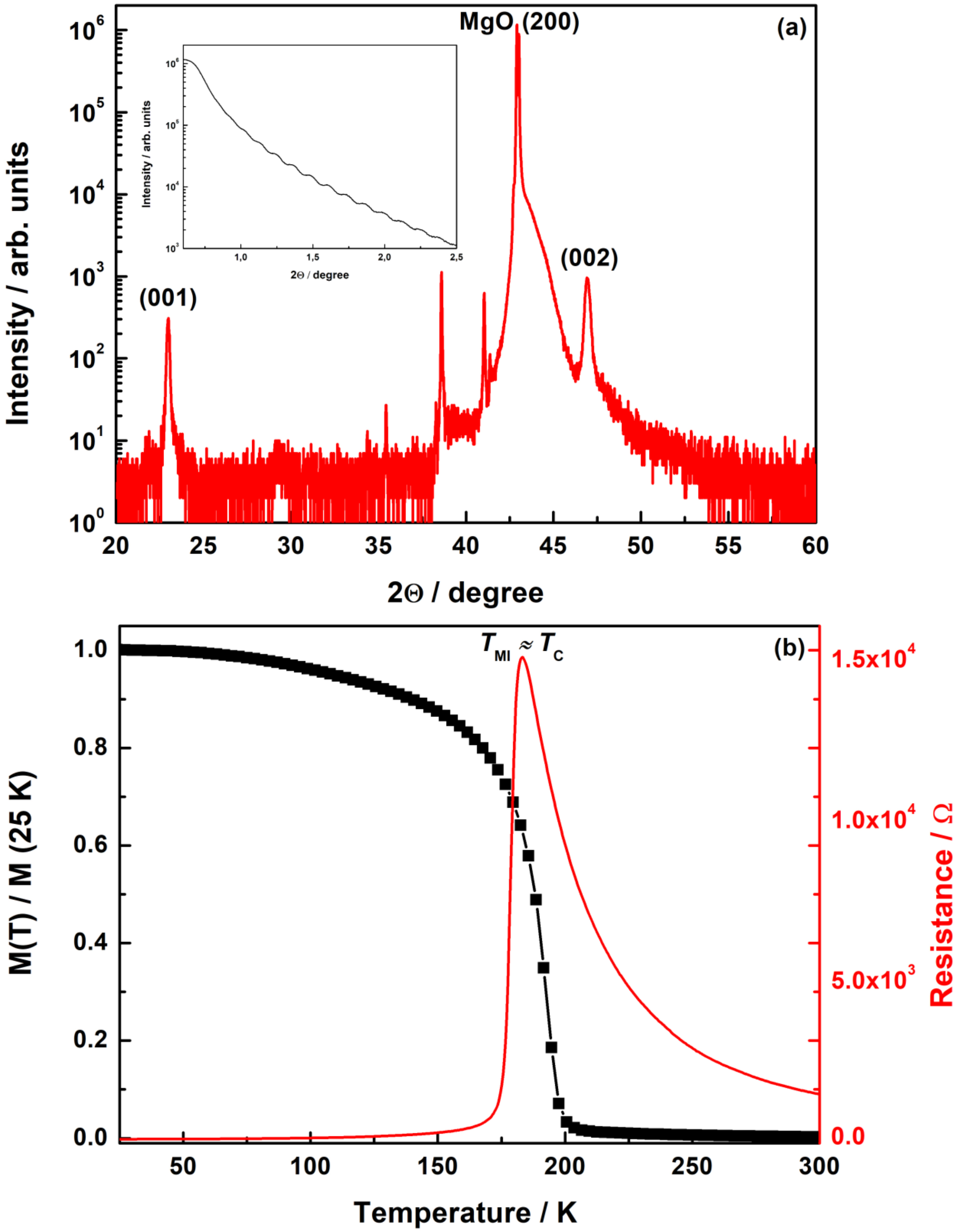

Fig. S1.2: $R(T)$ for different applied magnetic fields, H = 0 kOe, 10 kOe, 30 kOe and 50 kOe.

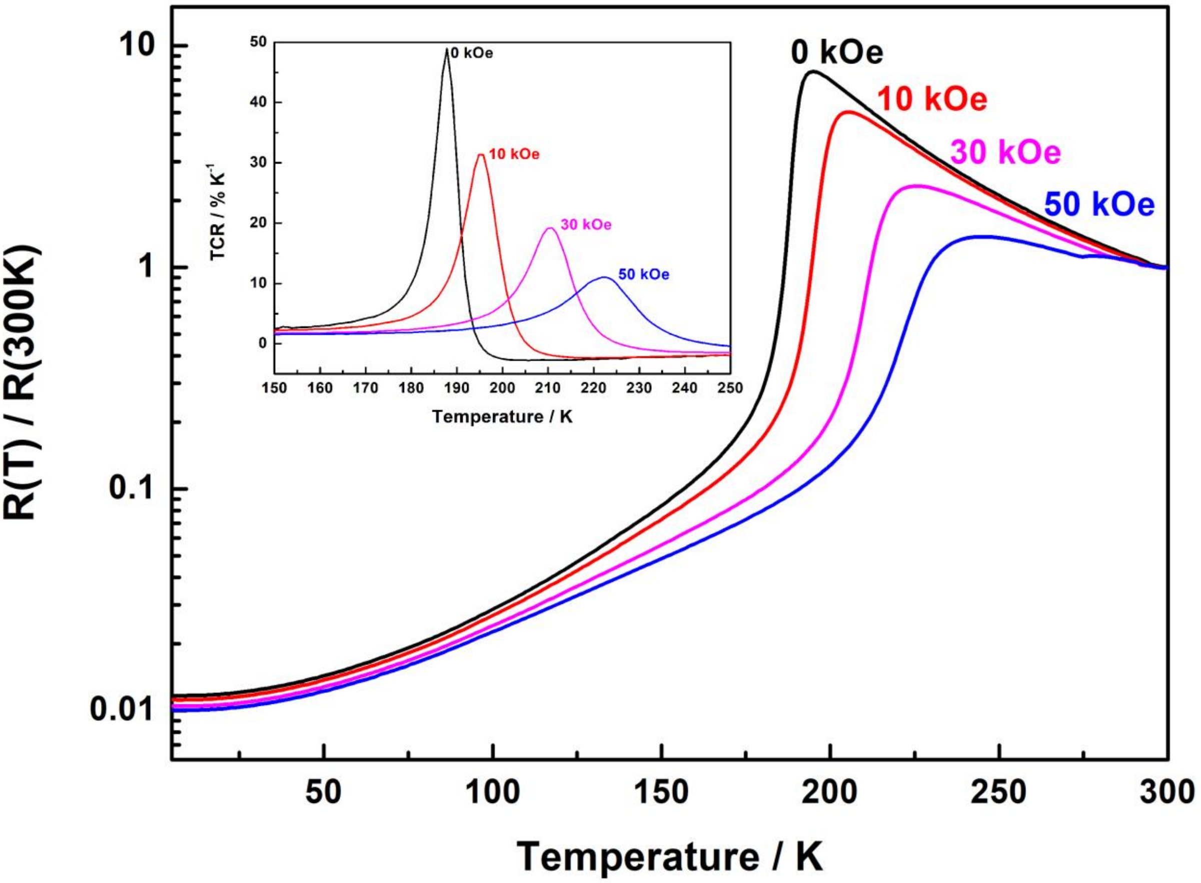

S2. Details of the evaluation of the Raman spectra of LPCMO

First, the Raman spectra were corrected by the Bose-Einstein thermal factor $(1 + n(\omega)) = [1 - \exp(-h\omega/k_BT)]^{-1}$. The electronic background continuum was modeled by a collision-limited model $R_{el} = BG\omega/(G^2+\omega^2)$ with B, G and ω as intensity, line width and frequency, respectively. The phonon peaks were modeled by the best fit of multiple Lorentzian line shapes.

Fig. S2.1: Power dependence of the line width of the 240 cm^{-1} mode at room temperature. For $P < 7.5$ mW, the line width practically does not change, indicating no significant laser-induced heating of the film.

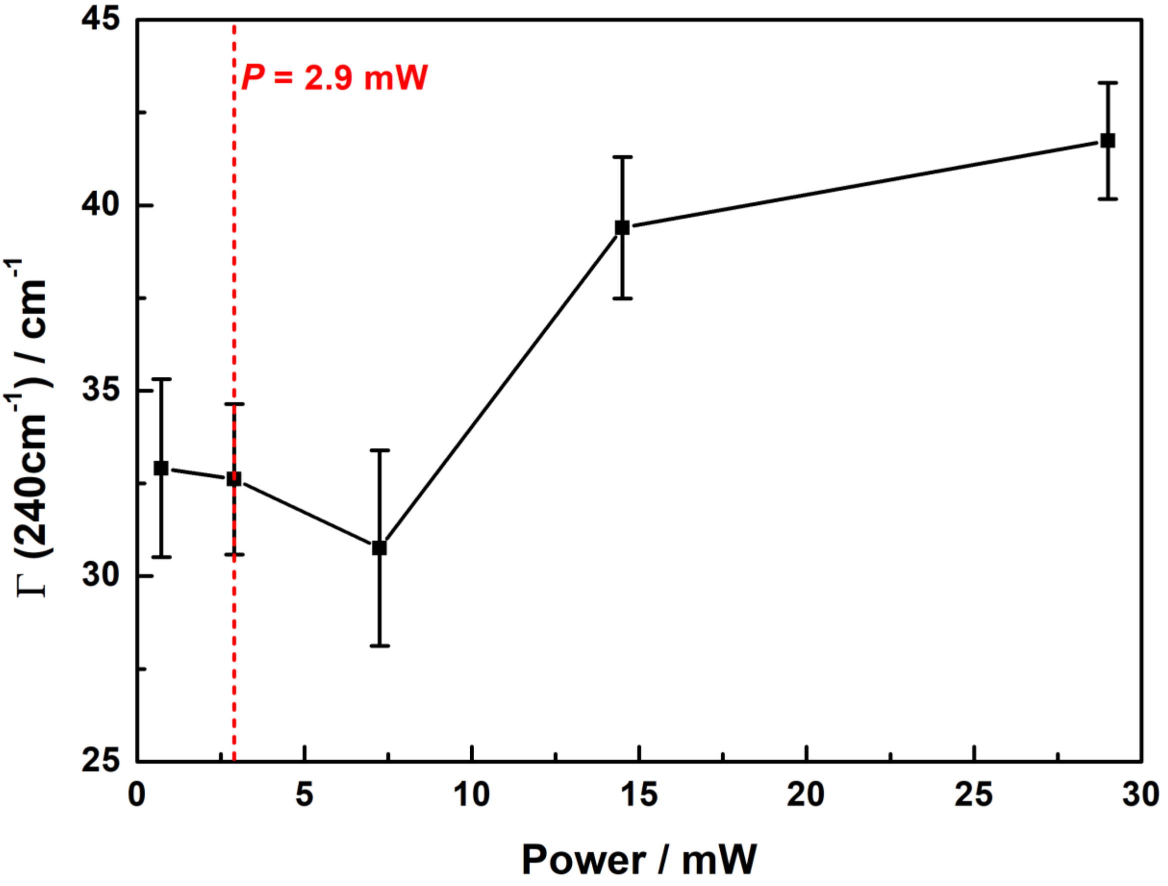

Fig. S2.2: Raw Raman spectra (black), the substracted baseline (dashed blue), the best fit of the Lorentzian line shapes (dotted green) and the resulting modeled Raman spectrum (red) of the LPCMO film for *H* = 0 kOe, 50 kOe at *T* = 195 K in the region of interest (300 – 1600 cm^{-1}).

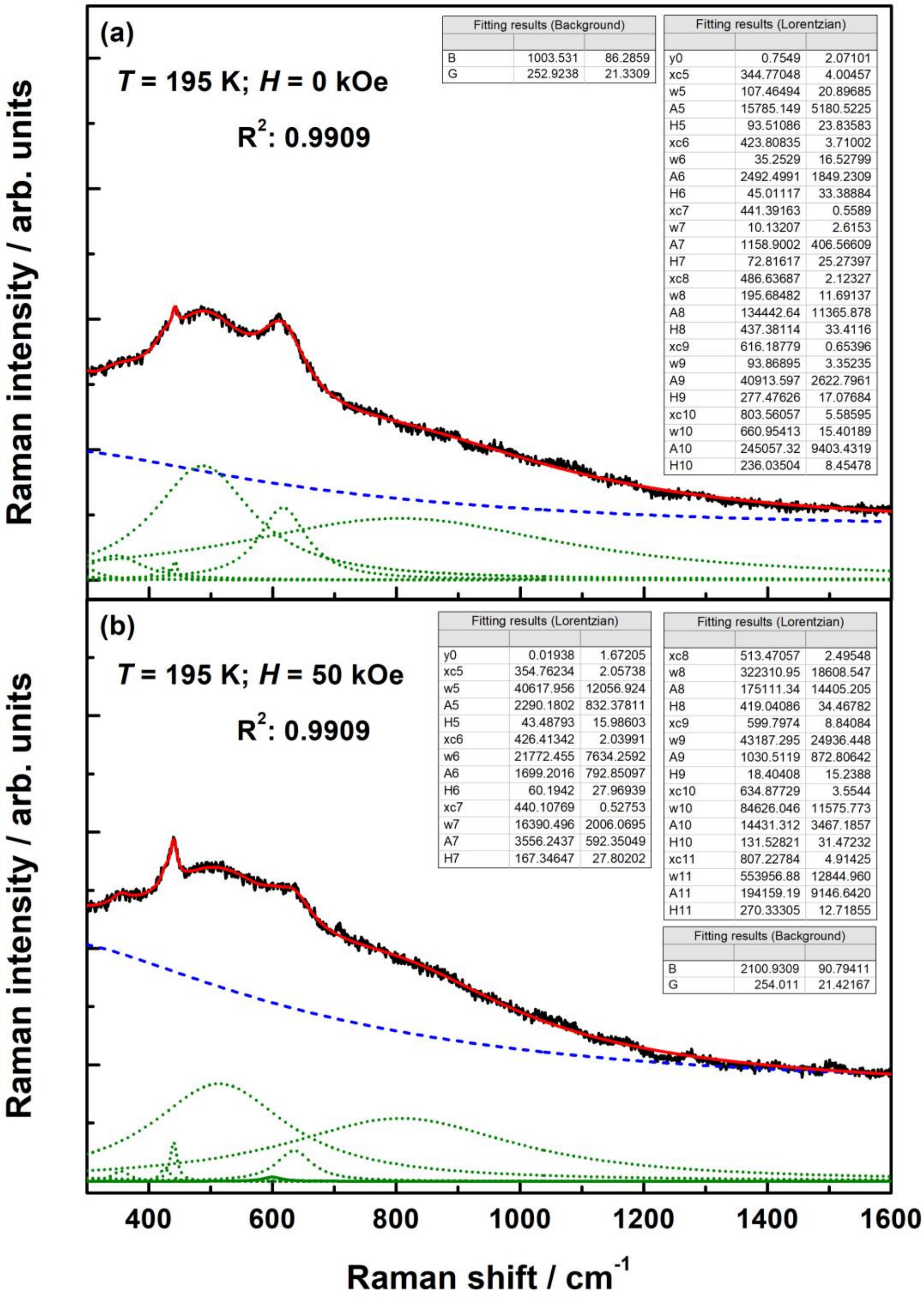

Fig. S2.3: Best fit of Lorentzian line shapes of the Raman spectra for (a) T = 150 K, (b) T = 195 K, (c) T = 245 K and (d) T = 275 K in the region of interest (300 – 900 cm^{-1}) for several temperatures of our temperature-dependent Raman study, presented in Fig. 4 in the main manuscript.

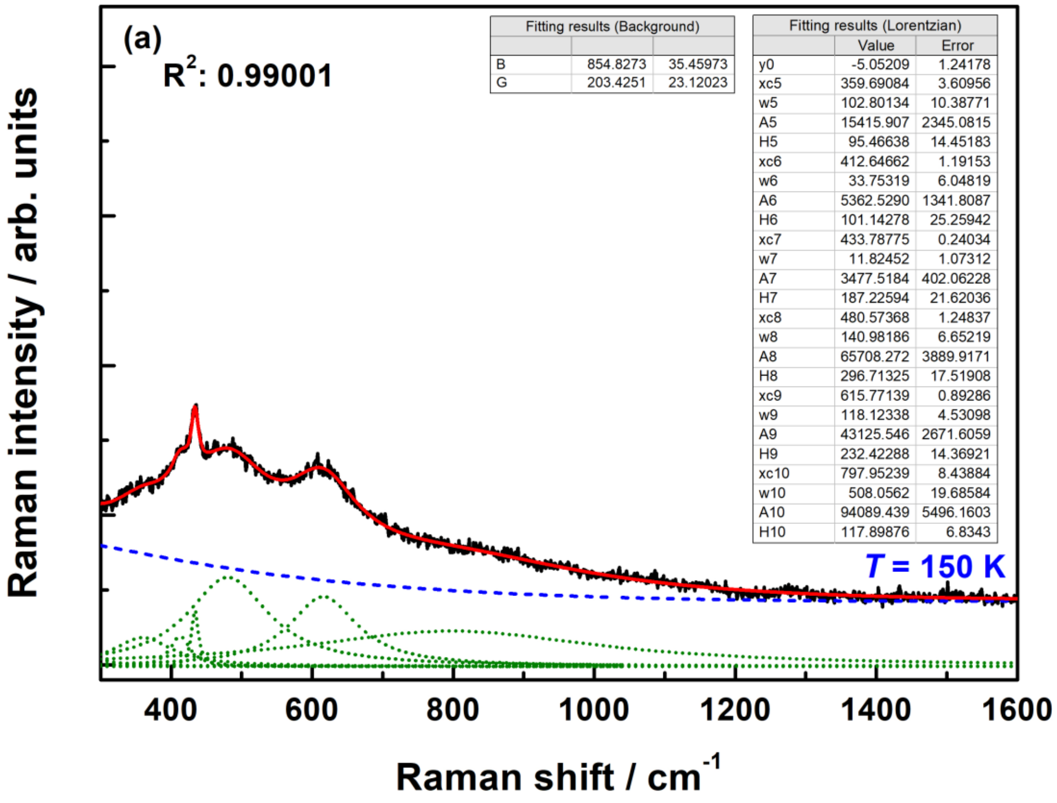

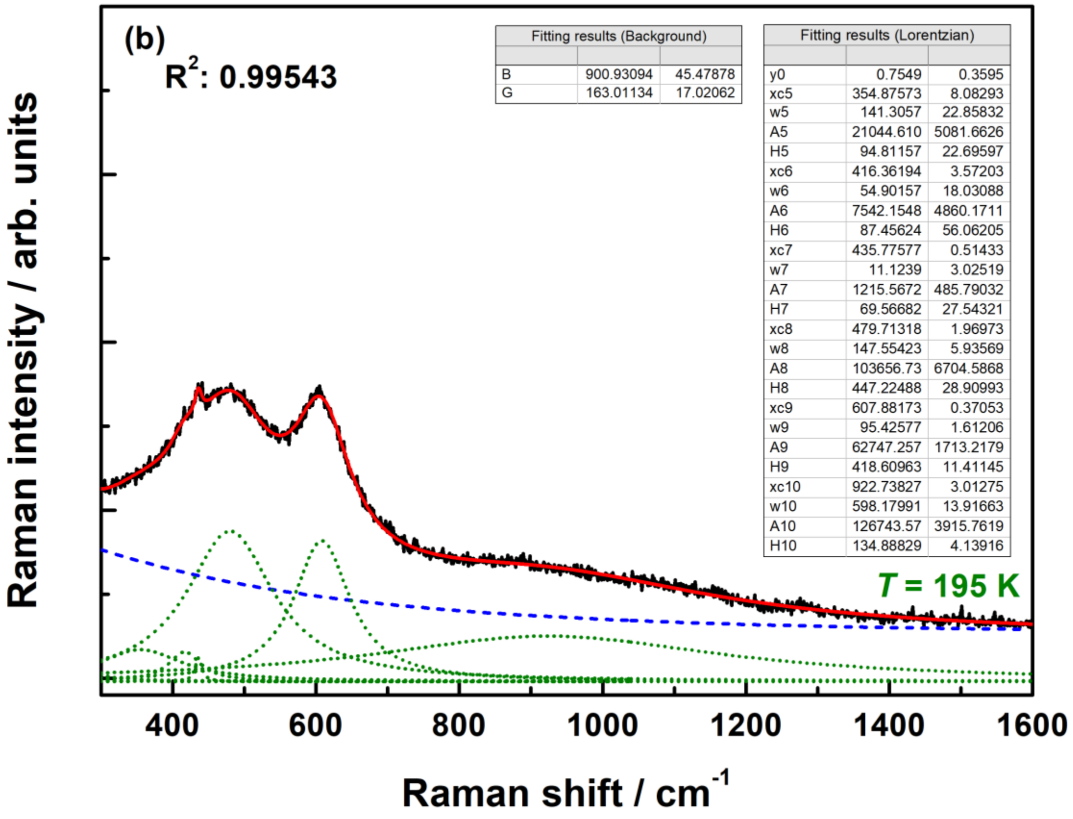

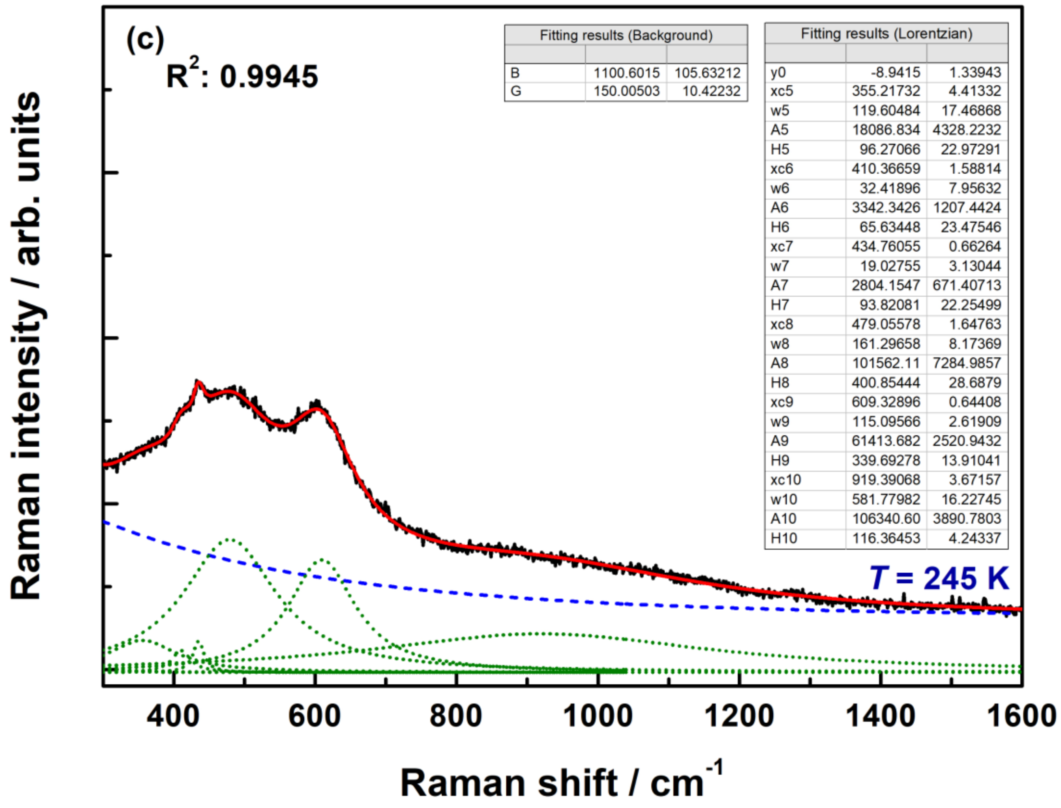
(c)
R²: 0.9945
Fitting results (Background)
B 1100.6015 105.63212
G 150.00503 10.42232
Fitting results (Lorentzian)
y0 -8.9415 1.33943
xc5 355.21732 4.41332
w5 119.60484 17.46868
A5 18086.834 4328.2232
H5 96.27066 22.97291
xc6 410.36659 1.58814
w6 32.41896 7.95632
A6 3342.3426 1207.4424
H6 65.63448 23.47546
xc7 434.76055 0.66264
w7 19.02755 3.13044
A7 2804.1547 671.40713
H7 93.82081 22.25499
xc8 479.05578 1.64763
w8 161.29658 8.17369
A8 101562.11 7284.9857
H8 400.85444 28.6879
xc9 609.32896 0.64408
w9 115.09566 2.61909
A9 61413.682 2520.9432
H9 339.69278 13.91041
xc10 919.39068 3.67157
w10 581.77982 16.22745
A10 106340.60 3890.7803
H10 116.36453 4.24337
T = 245 K
Raman intensity / arb. units
400
600
800
1000
1200
1400
1600
Raman shift / cm-1

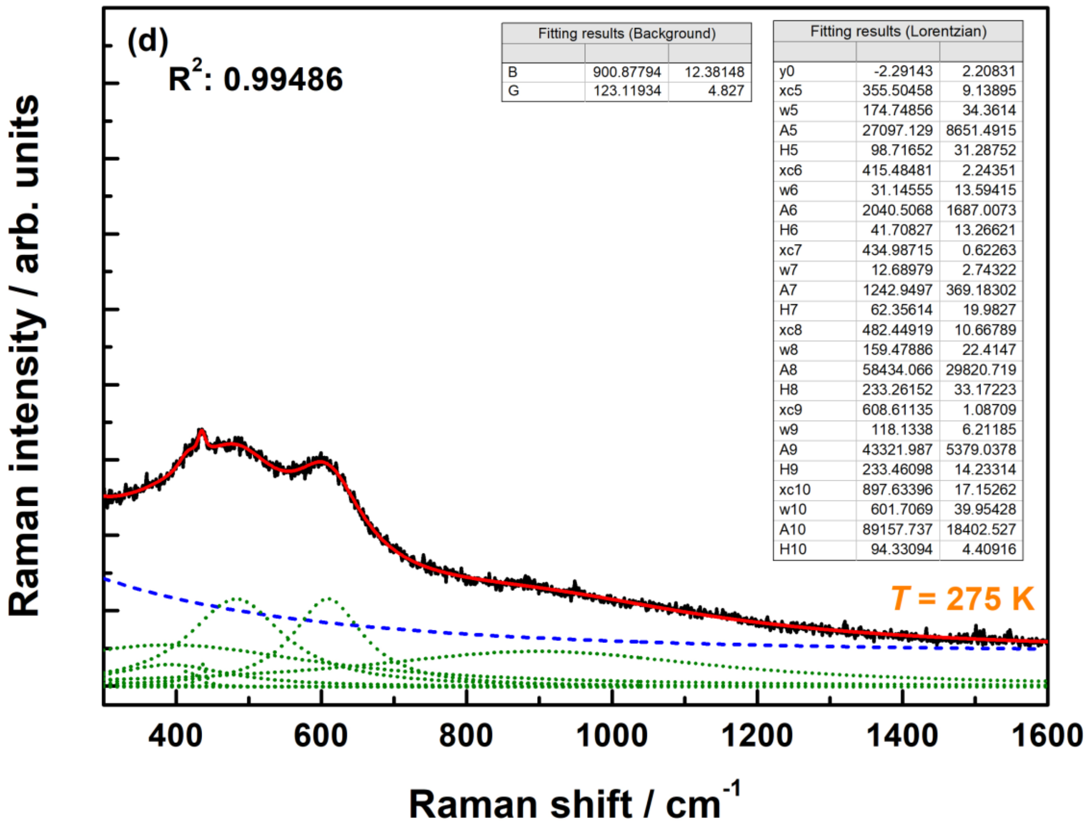
(d)
R²: 0.99486
Fitting results (Background)
B 900.87794 12.38148
G 123.11934 4.827
Fitting results (Lorentzian)
y0 -2.29143 2.20831
xc5 355.50458 9.13895
w5 174.74856 34.3614
A5 27097.129 8651.4915
H5 98.71652 31.28752
xc6 415.48481 2.24351
w6 31.14555 13.59415
A6 2040.5068 1687.0073
H6 41.70827 13.26621
xc7 434.98715 0.62263
w7 12.68979 2.74322
A7 1242.9497 369.18302
H7 62.35614 19.9827
xc8 482.44919 10.66789
w8 159.47886 22.4147
A8 58434.066 29820.719
H8 233.26152 33.17223
xc9 608.61135 1.08709
w9 118.1338 6.21185
A9 43321.987 5379.0378
H9 233.46098 14.23314
xc10 897.63396 17.15262
w10 601.7069 39.95428
A10 89157.737 18402.527
H10 94.33094 4.40916
T = 275 K
Raman intensity / arb. units
400
600
800
1000
1200
1400
1600
Raman shift / cm-1

Fig. S2.4: The splitting of the JT mode is also seen for $T < T_C$ and an applied magnetic field, H = 50 kOe.

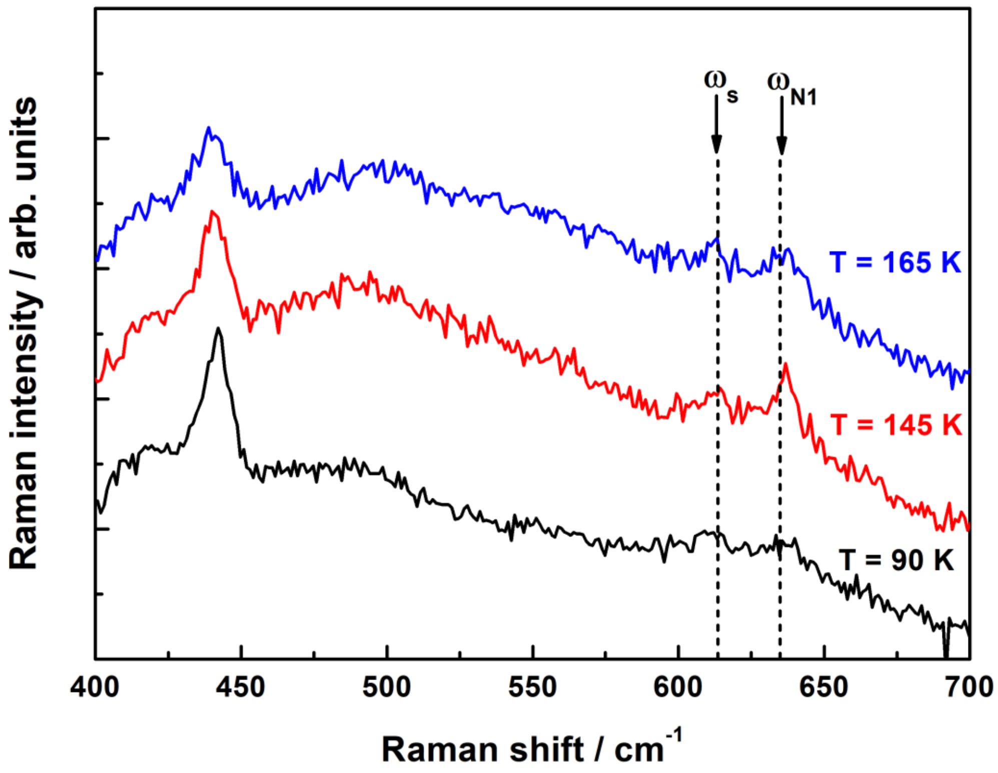

S3. Deduction of the T-H phase diagram from the M(H) curves

To create the *T*-*H* phase diagram, we measured the *M*(*H*) curves and reconstructed the *M*(*T*) curves by picking the magnetization value from the *M*(*H*) curve at different magnetic fields at a specific temperature. The obtained *M*(*T*) curves are shown in Fig. S3.1a. The $T_C(H)$ value of the *T*-*H* phase diagram is defined by the linear approximation of the *M*(*T*) curve to M = 0 emu/cm^3. The $T^*(H)$ is obtained from the field of a maximum of $dM/dH|_{T>Tc(0)}$. The *M*(*H*) curves and the corresponding $dM/dH|_{T>Tc(0)}$ curves are shown in Fig. S3.1b-c as well as in Fig. 4a in the main manuscript.

Fig.3.1: (a) The reconstructed *M*(*T*) curves for applied magnetic fields H = 0.1, 5, 10, 30 and 50 kOe. Exemplary, the derived transition temperatures $T_C(H)$ for H = 0.1 kOe and H = 50 kOe are marked by arrows. (b) Exemplary *M*(*H*) and (c) $dM/dH|_{T>Tc(0)}$ curves, from which we obtained the temperature of the metamagnetic transition, $T^*(H)$.

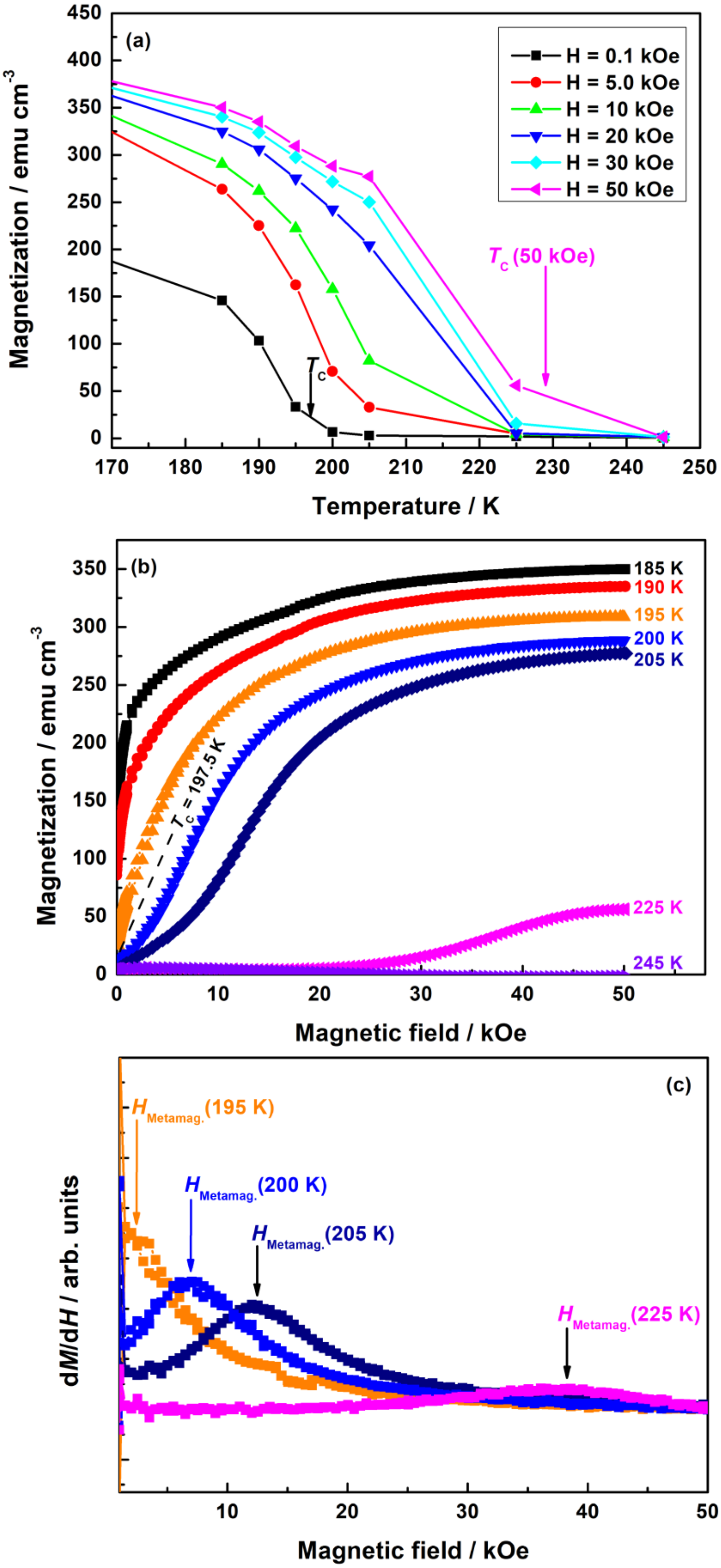
(a)
Magnetization / emu cm-3
Temperature / K
H = 0.1 kOe
H = 5.0 kOe
H = 10 kOe
H = 20 kOe
H = 30 kOe
H = 50 kOe
TC (50 kOe)
TC
(b)
Magnetization / emu cm-3
Magnetic field / kOe
185 K
190 K
195 K
200 K
205 K
225 K
245 K
TC = 197.5 K
(c)
dM/dH / arb. units
Magnetic field / kOe
HMetamag.(195 K)
HMetamag.(200 K)
HMetamag.(205 K)
HMetamag.(225 K)

S4. Field alignment in the optical setup

Figure S4 shows a sketch of the optical experiment. The laser beam was focused onto the sample by using a 50xLWD objective with a working distance, d_{WD} = 10.6 mm. The sample was mounted on a Cu sample holder (thermal contact with silver paste at the corners of the sample), which is placed in the center of a superconducting solenoid magnetic coil inside a commercial cryostat (Microstat MO, *Oxford Instruments*) in vacuum (10^{6}-10^{7} mbar). The field homogeneity is below 2% over a 5 mm diameter sphere with stability is 0.01%/h [1]. The field can be considered as homogenous inside the probed sample area, since the Raman spectrometer only probes a small area of the sample (< 2 μm).

Fig. S4: Sketch of the alignment of the optical experiment and the field alignment in the cryostat.

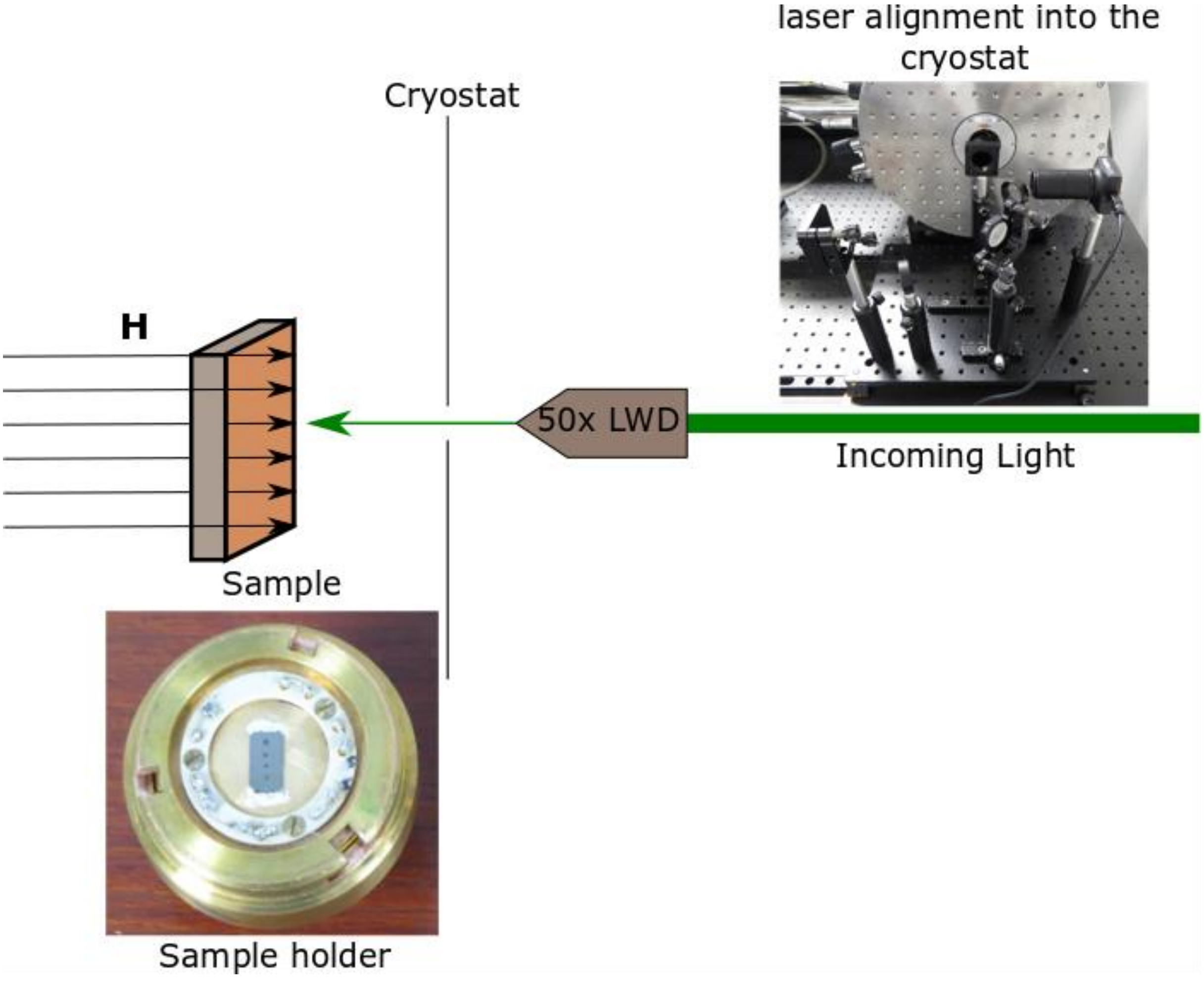

References

[1] Product information of the "Microstat MO, *Oxford Instruments*" can be found at: http://www.labis.pl/resources/image/2015/karty/oxford/Microstat_MO.pdf.
